# Waning of Anti-SARS-CoV-2 Spike Antibody Levels 100 to 200 Days after the Second Dose of the BNT162b2 Vaccine

**DOI:** 10.3390/vaccines10020177

**Published:** 2022-01-24

**Authors:** Hidenori Matsunaga, Hidefumi Takeuchi, Yuichiro Oba, Satoshi Fujimi, Tomoyuki Honda, Keizo Tomonaga

**Affiliations:** 1 Department of Psychiatry, Osaka General Medical Center, Osaka 558-8558, Japan; 2 Institute for General Research, Nihon Igaku Ltd., Kaizuka-City, Osaka 597-0081, Japan; takeuchi@jml-group.co.jp; 3 Department of General Medicine, Osaka General Medical Center, Osaka 558-8558, Japan; ooba@mbj.ocn.ne.jp; 4 Division of Trauma and Surgical Critical Care, Osaka General Medical Center, Osaka 558-8558, Japan; fujimis@opho.jp; 5 Department of Virology, Graduate School of Medicine, Dentistry and Pharmaceutical Science, Okayama University, Okayama 700-8558, Japan; thonda@virus.med.osaka-u.ac.jp; 6 Laboratory of RNA Viruses, Department of Virus Research, Institute for Frontier Life and Medical Sciences, Kyoto University, Kyoto 606-8507, Japan; tomonaga.keizo.5r@kyoto-u.ac.jp

**Keywords:** anti-SARS-CoV-2 spike antibody, BNT162b2 vaccine, sex-associated differences, third antigen stimulation, delta variant

## Abstract

Anti-SARS-CoV-2 antibodies of 444 vaccinated hospital employees in Japan were measured 94–109 days and 199–212 days after receiving the second BNT162b2 vaccine dose to evaluate the intensity and duration of antibody response in our own cohort. Among uninfected participants, anti-S antibody levels were greatly decreased 199–212 days after the second vaccination compared to the levels measured 94–109 days after the second vaccination (median levels: 830 AU/mL and 2425 AU/mL, respectively; *p* < 0.001). The rate of decrease between the two testing periods was lower in infected participants than in uninfected participants (median: 47.7% and 33.9%, respectively; *p* < 0.001). Anti-S antibody levels were significantly higher in females (median: females, 2546 AU/mL; males, 2041 AU/mL; *p* = 0.002 during the first test period). The peak body temperature after vaccination was higher in females than in males (median: females, 37.4 °C; males: 37.1 °C; *p* = 0.044). Older males tended to have lower antibody levels. In conclusion, the duration of the anti-S antibody response to the BNT162b2 vaccine was short-lived, particularly in males. Anti-S antibody levels of 1000 AU/mL or lower according to SARS-CoV-2 IgG II Quant (Abbott) might indicate insufficient prevention against the delta variant, and the majority of participants appeared to have lost their protection 200 days after vaccination.

## 1. Introduction

Vaccines are expected to become the most effective method of controlling the coronavirus disease (COVID-19) pandemic. Vaccination started in December 2020 in Israel; however, the effectiveness of the vaccines was reported to decrease within 6 months of administration [1]. Indeed, the number of cases of breakthrough infection rapidly increased in July 2021 in Israel, and the health authorities started providing a third dose of the vaccine on 1 August 2021. Other countries have also adopted the same procedure.

In Japan, five epidemic waves occurred in 2020 and 2021: in April 2020, from July to August 2020, from November 2020 to January 2021, from April to May 2021, and from July to September 2021. Approximately 1.7 million people were infected, and there were 18,000 COVID-19 deaths by the end of December 2021. The BNT162b2 vaccine was approved on 14 February 2021, and vaccination of hospital workers started on 17 February 2021. In our hospital, vaccination started on 8 March 2021. Vaccination of the general population older than 65 years started on 12 April 2021, and to those aged 12 to 64 years in June 2021. Eighty percent of older people had received two doses of the vaccine before the end of July 2021, and the proportion of older people among those with severe COVID-19 was markedly decreased during the fifth wave from July to September 2021. Overall, 76.9% of the entire population had received two doses of the vaccine by the end of November 2021 (https://www.kantei.go.jp/jp/headline/kansensho/vaccine_supply.html, accessed on 5 January 2022). In Japan, the mRNA 1273 and Ad26.COV2.S COVID-19 vaccines were approved on 17 May 2021. BNT162b2 and mRNA-1273 are the main COVID-19 vaccines used in Japan.

One cause of decreased vaccine effectiveness might be the short duration of the vaccine-induced antibodies. Second, vaccines may be less effective against the SARS-CoV-2 delta variant, which caused an epidemic in India in April 2021 and spread worldwide within two months [2]. The efficacy of the vaccines was strictly examined in the manufacturers’ clinical studies and further evaluated by nationwide large-scale studies. Utilizing data obtained in such ways, governments have decided and implemented the schedules of vaccination. The aim of this study was to obtain a better understanding of the effectiveness and limitations of COVID-19 vaccines and the dynamics of the antibody production in our own cohort.

## 2. Materials and Methods

### 2.1. Participants and Setting

**Figure 1:**
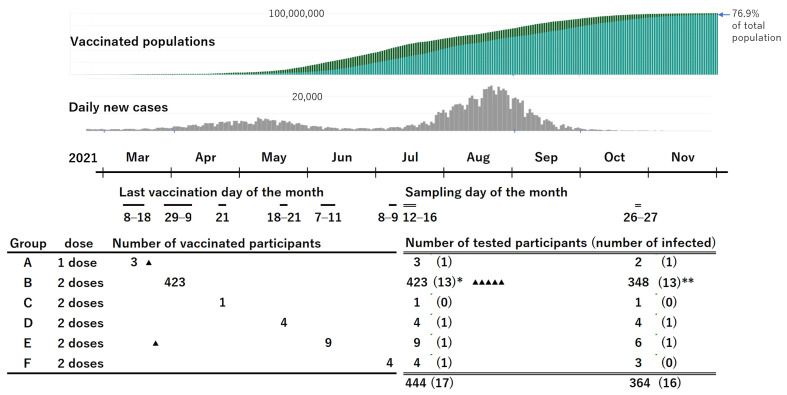
Vaccinated populations, daily new cases in Japan, and schedules of vaccination and blood sampling in the study participants. “▲” indicates participants who had SARS-CoV-2 infection confirmed by PCR after vaccination. The triangle also indicates the timing of the diagnosis. * The number in the parentheses includes 11 participants with a history of positive PCR results and 2 with positive anti-N antibody results without PCR positive results. ** The number in the parentheses includes 7 participants with a history of positive PCR results, 1 with a history of positive anti-N antibody results without PCR positive results, and 5 newly infected and confirmed by PCR after the first testing. (https://news.yahoo.co.jp/pages/article/20200207, accessed on 6 January 2022; https://www.worldometers.info/coronavirus/country/japan/, accessed on 6 January 2022).

This study included employees of Osaka General Medical Center in Osaka City, Japan. The facility is a tertiary-level hospital with 865 beds and several departments, including an emergency unit. Of the 2000 employees, 1550 received the first dose of the BNT162b2 vaccine between 8 March and 19 March 2021; they received the second dose between 29 March and 9 April 2021. A few other employees received the vaccination according to delayed schedules, as described in Figure 1. No vaccines other than BNT162b2 (Pfizer Inc, New York, NY, USA) were used. All employees who had received at least one dose of the BNT162b2 vaccine and agreed to provide a blood sample were eligible for inclusion in the study.

### 2.2. Data Collection and Sample Collection, Transport, and Storage

Participants were screened for COVID-19 using anti-SARS-CoV-2 nucleocapsid protein (anti-N) antibody tests. Polymerase chain reaction (PCR) testing for SARS-CoV-2 infection was not performed, but the employees were tested whenever they were suspected of having COVID-19. Participants were questioned about COVID-19 symptoms, previous PCR-confirmed COVID-19, and any side effects they experienced after they were vaccinated. They were not asked about self-antigen test results because these tests are not common in Japan.

During a routine general medical examination, a 5 mL blood sample was collected from each participant in a serum-separator tube between 12 July and 16 July 2021. Between 26 October and 27 October 2021, a blood sample was collected from each participant for follow-up measurements. The samples were immediately stored at 4 °C and transported to the research laboratory, where the anti-S antibodies were measured the following day. The residual samples were stored at 4 °C and transported to the radioisotope laboratory at the Graduate School of Medicine of Osaka University, where they were tested for anti-N antibodies, 8 to 12 days after sampling. The samples were stored at 4 °C from the time of sampling until test completion.

### 2.3. Ethics and Informed Consent

This research was approved by the Ethics Committee of Osaka General Medical Center (approval number: 2021-023). Written informed consent was obtained from all participants.

### 2.4. Laboratory Methods

Anti-S antibodies were measured using SARS-CoV-2 IgG II Quant (Abbott Diagnostics, Chicago, IL, USA) according to the manufacturer’s instructions, and values >50 AU/mL were considered positive.

Anti-N antibodies were measured using an in-house radioligand assay by one of the authors [3]. Briefly, cDNA encoding SARS-CoV-2 N with a His-tag and T7-tag at the N-terminal were inserted into pET28a. Radiolabeled nucleocapsid antigens were produced by incubating a mixture containing the plasmid, an in vitro transcription/translation kit, and ^35^S-methionine at 30 °C for 90 min. Radiolabeled nucleocapsid protein was separated using a column. Then, 96-well filter plates were used as containers for the 50 µL reaction mixture, including 1 µL of serum. Human IgG-absorbing resin was added to precipitate the antigen–antibody complex. After overnight incubation, the precipitate was washed for 1 h. After drying, the scintillation cocktail was added, and radioactivity was measured. The index value was determined as follows: the average of the negative samples in the 96-well plate, excluding those with high or slightly high values, was considered to have an index value of 0, and the average from three positive controls with different antibody levels was determined to have an index value of 8. Using 49 samples from inpatients who had recovered from COVID-19, we found that the sensitivities with the cut-off value of 5.0, 4.0, 3.0, and 2.0 were 91.8%, 95.9%, 100%, and 100%, respectively; using 500 stored sera taken before the pandemic, the specificities were 100%, 99.4%, 99.2%, and 98.8%, respectively. In addition, the results of 200 samples by the radioligand assay were compared with the results of a chemiluminescent immunoassay, performed using an automatic analyzer (iFlashTM, YHLO Biotechnology Company Ltd., Shenzhen, China) and found to have very good correlation [3]. On the basis of these results, we considered index values >5.0 positive, and values between 2.0 and 5.0 were considered borderline.

The PCR test was able to detect all variants prevailing in Japan, including the delta variant.

### 2.5. Statistical Analysis

The results were summarized using frequencies and percentages for categorical variables; medians and interquartile ranges (IQRs) for continuous variables. The statistical significance of the differences in anti-S antibody levels between groups was assessed using the Mann–Whitney *U* test. The correlations of anti-S antibody levels with age and body temperature were assessed using Spearman’s rank correlation coefficient. SPSS version 27 (IBM Corp., Armonk, NY, USA) was used for the analyses. Two-sided *p*-values < 0.05 were considered statistically significant.

## 3. Results

A total of 444 employees were recruited, including 99 males aged 24–65 years (median: 39 years; IQR: 31–54 years) and 345 females aged 22–64 years (median: 36 years; IQR: 27–50 years). The schedules of vaccination including the doses and the number of participants for each schedule are shown in Figure 1. Group B was the main cohort, and the others were used to help evaluate the results of group B. The interval between the administration of the second dose of the vaccine and blood sampling for group B was 94 to 109 days (median: 102 days; IQR: 98–105 days).

A second blood sample was obtained from 364 of 444 participants (82%), including 348 participants in group B. The interval between the second vaccination and the second blood sampling for participants in group B was 199 to 212 days (median: 207 days; IQR: 203–209 days).

At the time of the first blood sampling, 15 participants had a history of COVID-19 confirmed using PCR, 13 of whom were infected before their first vaccination. Two participants were infected with SARS-CoV-2 in March 2021, approximately one week after the first vaccination. One of them in Group E received the second vaccination on 7 June 2021. All the other participants in Groups B–F had received 2 doses of the vaccine with an interval of 3 weeks. Among the 15 participants with past infection detected in the first testing, 10 participated in the second sampling. In addition, another five participants were found to be newly infected with SARS-CoV-2 after the first blood sample was obtained. Another 27 participants had possible infection because of having some symptoms or close contact with someone with COVID-19; however, the PCR test results were all negative. Moreover, all of them were negative for anti-N antibodies, except for one with a borderline result.

### 3.1. Anti-SARS-CoV-2 Nucleocapsid Protein Antibodies

**Figure 2:**
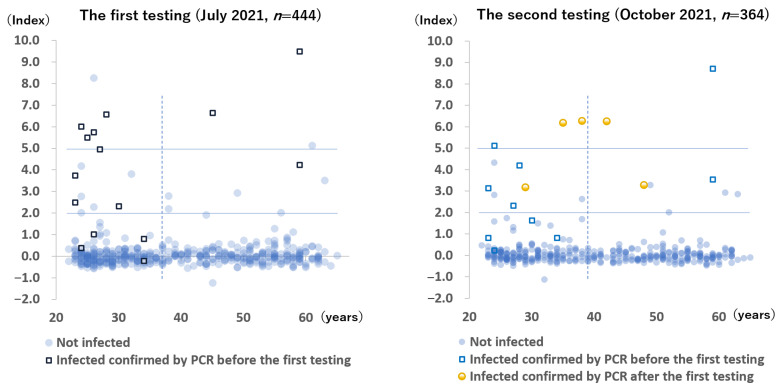
Anti-SARS-CoV-2 nucleocapsid protein antibody levels of all participants. The *x*-axis indicates age. The solid lines indicate the range of the borderline positive antibody levels. The dotted line indicates the median age of the participants (37 years during the first testing period and 39 years during the second testing period).

The anti-N antibody results of all participants during the two testing periods are shown in Figure 2. During the first testing period, 94 to 109 days after the last vaccination, 8 (1.8%) blood samples were positive, 15 (3.4%) blood samples were borderline positive, and 421 (94.8%) blood samples were negative. Conversely, among the 15 participants with active PCR-confirmed COVID-19, 6 had positive results, 5 had borderline positive results, and 4 had negative results. Two participants without a history of COVID-19 had positive results for anti-N antibody—one was suspected to be infected with SARS-CoV-2, 3 weeks after the second vaccination when she came into close contact with a patient with COVID-19, whereas the other had no symptoms and no identifiable source of infection.

During the follow-up testing period, 5 (1.4%) samples were positive, 13 (3.6%) samples were borderline positive, and 346 (95.1%) samples were negative. Among the 5 newly infected participants after the first testing period, 3 had positive results, and 2 had borderline positive results.

### 3.2. Anti-SARS-CoV-2 Spike Protein Antibody Levels

In evaluating the anti-S antibody levels, we found that participants with a history of either positive PCR results or positive anti-N antibody results were considered as having a history of infection. Participants with borderline results for anti-N antibody and without PCR positive results were considered not to be infected in order to exclude false-positive assessments.

**Figure 3:**
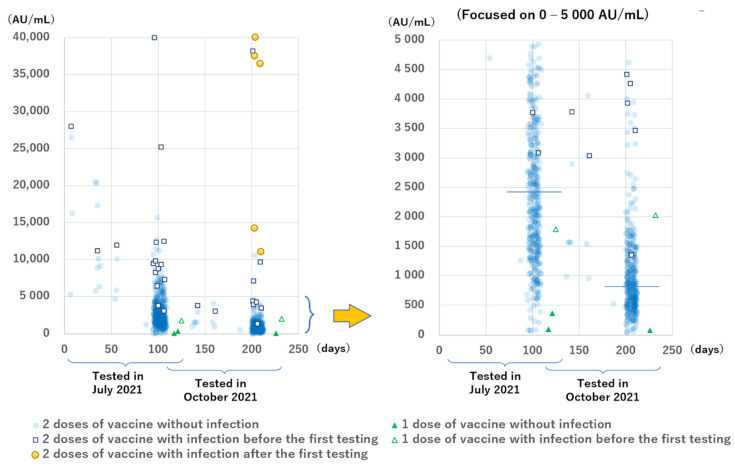
Anti-SARS-CoV-2 spike protein antibody levels of all participants according to the time since the most recent dose of the BNT162b2 vaccine was administered. The horizontal line indicates the median levels among participants without infection tested 94 to 109 days (2425 AU/mL) and 199 to 212 days (813 AU/mL) after the second vaccination. Infection includes PCR positive results and/or anti-N antibody positive results.

The anti-S antibody levels are shown in Figure 3. All the results except for two during the second testing period were positive, but the levels had a wide range. Participants with COVID-19 had higher levels than those without infection. The median anti-S antibody levels of the 13 infected and 410 non-infected participants in group B during the first testing period at 94 to 109 days after the second dose of the vaccine were 9324 AU/mL (IQR: 6859–12,397 AU/mL) and 2425 AU/mL (IQR: 1521–3641 AU/mL), respectively (*p* < 0.001). The median anti-S antibody levels of the 8 infected participants, except the 5 newly infected ones and 335 non-infected participants in group B during the second testing period at 199 to 212 days after the second dose of vaccination were 4339 AU/mL (IQR: 3698–8389 AU/mL) and 813 AU/mL (IQR: 519–1321 AU/mL), respectively (*p* < 0.001).

Focusing on the antibody levels with a shorter interval between vaccination and sampling, we found that 12 of the 13 (92%) participants without infection in groups D, E, and F who were tested at 6 to 56 days after the second vaccination had anti-S antibody levels >5000 AU/mL, whereas only 54 of 410 participants (13.2%) without infection in group B who were tested at 94 to 109 days after the second vaccination had anti-S antibody levels >5000 AU/mL.

Regarding the number of the doses, two participants without infection in group A tested at 117 to 121 days after receiving one vaccine had low levels of anti-S antibodies (96 AU/mL and 364 AU/mL); furthermore, one participant tested at 125 days after receiving one vaccine, who had an active infection (PCR positive and anti-N antibody borderline positive results) 6 days after receiving the first vaccine, had an anti-S antibody level of 1789 AU/mL.

**Figure 4:**
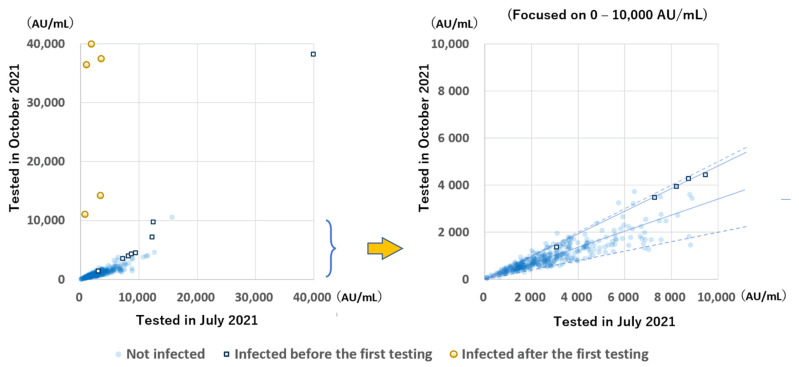
Relation between the anti-S antibody levels tested, 94 to 109 days and 199 to 212 days after the second vaccination. The solid line indicates the median decrease in participants with and without infection (47.7% and 33.9%, respectively). Dotted lines indicate 50% and 20%. Most participants had antibody levels within the 20–50% range.

The anti-S antibody levels during the two testing periods of the 348 participants in group B were compared (Figure 4). A great increase was observed in 5 participants infected after the initial sampling. In almost all the remaining participants, except for one participant with very low antibody levels, the antibody levels were greatly decreased, mostly to 20–50%, while the rate of decrease of the antibody levels between the two testing periods in infected participants was more gradual than in uninfected (median: 47.7% and 33.9%, respectively; *p* < 0.001).

**Figure 5:**
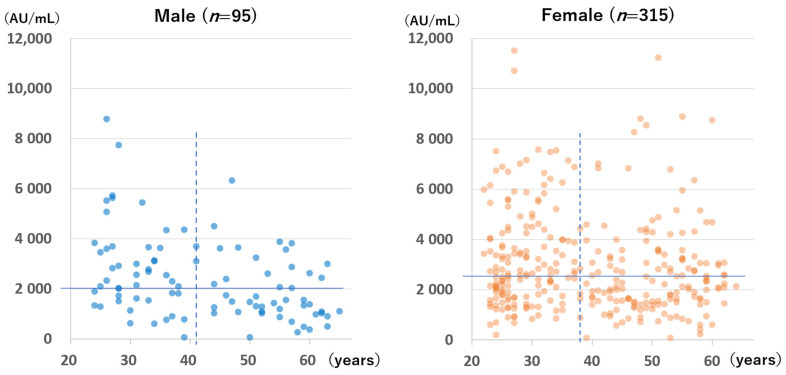
Anti-spike antibody levels of participants without a history of SARS-CoV-2 infection at 94 to 109 days after the second dose of the BNT162b2 vaccine according to sex and age. The horizontal lines indicate the median antibody levels of each group (males: 2043 AU/mL; females: 2546 AU/mL). The perpendicular dotted lines indicate the median age of each group (males: 41 years; females: 38 years).

The anti-S antibody levels of the 410 participants without infection in group B who were tested during the first testing period are shown according to sex and age in Figure 5. Females had significantly higher antibody levels than males (median for females: 2546 AU/mL; median for males: 2043 AU/mL; *p* = 0.002). However, a significant difference was not observed for those tested at 199 to 212 days after the second vaccination (median for females: 845 AU/mL; median for males: 712 AU/mL; *p* = 0.239; figure not shown).

A significant negative correlation was found between age and anti-S antibody level for males but not for females at the time of the first measurement (Spearman’s rank correlation coefficient for males: −0.40, *p* < 0.001; Spearman’s rank correlation coefficient for females: −0.076, *p* = 0.174) and at the time of the second measurement (Spearman’s rank correlation coefficient for males: −0.48, *p* < 0.001; for females: −0.10, *p* = 0.09).

### 3.3. Highest Body Temperatures after Vaccination

**Figure 6:**
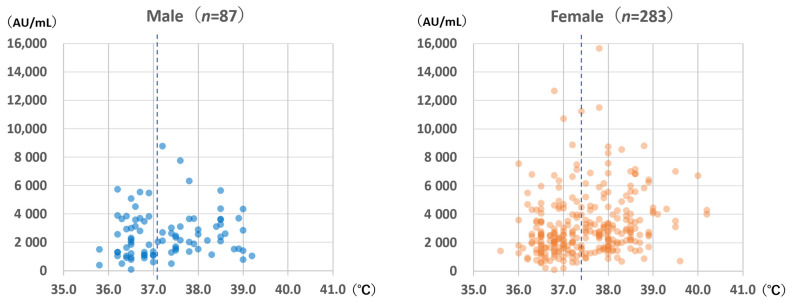
Anti-spike antibody level according to sex and highest body temperature within 1 week after vaccination with the BNT162b2 vaccine. The dotted lines indicate the median body temperature values (males: 37.1 °C; females: 37.4 °C).

The highest body temperatures within 1 week after vaccination are shown in Figure 6. Females experienced significantly higher body temperatures than males (median for females: 37.4 °C; median for males: 37.1 °C; Mann–Whitney *U* test: *p* = 0.044). For females, body temperature was significantly correlated with anti-S antibody levels (Spearman’s rank correlation coefficient: 0.253; *p* < 0.001); however, the same was not observed for males (Spearman’s rank correlation coefficient: 0.169; *p* = 0.12).

## 4. Discussion

Most infected participants with a history of COVID-19 and/or positive anti-N antibody results had high anti-S antibody levels. Among individuals with a history of COVID-19, the first dose of the vaccine provided a second antigen stimulus; therefore, this finding was expected. In contrast, participants who had received only one dose of the vaccine and had no history of COVID-19 had low anti-S antibody levels. The number of episodes of antigen stimulation is a key determinant of antibody levels.

The antibody levels of those tested at 6 to 56 days after the second vaccination were higher than those of participants tested 94 to 109 days after the second vaccination, suggesting that the anti-S antibodies produced by the vaccine had already decreased at 94 to 109 days after vaccination. The antibody levels retested approximately 100 days later were greatly decreased in all participants except those who were newly infected.

Sufficient levels of anti-S antibodies in the serum will combine with spike proteins of SARS-CoV-2 immediately after entering the host and can prevent infection. Therefore, the attenuation of antibodies should lead to a decrease in effectiveness. A study of the clinical efficacy of the BNT162b2 vaccine found that it prevented 95% of infections 90 days after the second vaccination [4]. A recently published clinical trial of the BNT162b2 vaccine found that the efficacy rates of preventing infection at 2, 4, and 6 months after the second vaccination were 96.2%, 90.1%, and 83.7%, respectively [1].

On the basis of the combined results of the two studies, we found that 5% of vaccinated individuals tested 90 days following BNT162b2, as well as 10% of those tested 4 months post-vaccination, should have levels of anti-S antibodies that appear inadequate to prevent infection. Among those tested at 94 to 109 days post-vaccination in our study, the 5th and 10th percentile levels were 710 and 940 AU/mL, respectively. Therefore, it appears that if the anti-S antibody level is <800 AU/mL when measured using the SARS-CoV-2 IgG II Quant assay, the BNT162b2 vaccine may not effectively prevent infection.

Five participants were newly infected between the first measurement and the second measurement. They all were infected in August, approximately 1 month after the first examination, and their anti-S antibody levels were greatly increased. Their anti-S antibody levels at the time of the first examination in July were 730 AU/mL, 1000 AU/mL, 1830 AU/mL, 3380 AU/mL, and 3520 AU/mL. Notably, three participants with relatively low anti-S antibody levels had acute symptoms for 7 to 10 days, followed by residual symptoms for 2 months; however, the other two participants with antibody levels higher than 3000 AU/mL were asymptomatic or had subtle symptoms for 5 days. These findings suggest that the severity and duration of the symptoms might be related to the antibody levels. Considering that the participant with an antibody level of 1830 AU/mL in July was infected in August and showed the full course of the symptoms, a level of approximately 1000 AU/mL might not be sufficient to prevent infection. In October, 199 to 212 days after the second vaccination, 62.8% of participants without infection had antibody levels <1000 AU/mL, and 82.1% had antibody levels <1500 AU/mL, which were projected to decrease to <1000 AU/mL within a few months.

Real-world mega-data analysis of the efficacy of the BNT162b vaccine according to the length after the second vaccination was performed in Israel [5]. Among the 13,426 fully vaccinated and newly confirmed COVID-19 cases between July 11 and 31, the rate of infection was increased in those who were vaccinated during March 16–31, 102–137 days after the second vaccination, or earlier. Therefore, the sampling point at 94 to 109 days after the second vaccination in this study should be the time just before the efficacy of the BNT162b vaccine began to decrease. This suggests that the antibody level of 1000 AU/mL might be an approximate indicator of the loss of effectiveness to prevent infection.

Among participants tested twice in group B, seven out of the 8 participants infected with SARS-CoV-2 during the first testing period had been infected prior to receiving the vaccine, showing higher anti-S antibody levels than those without infection and lower rates of decrease of the antibody levels. These findings suggest that the anti-S antibody levels after the third stimulation with antigens, such as the third vaccination, may be higher and more persistent than those after the second stimulation with antigens.

One of the two participants who was anti-N antibody-positive without a history of obvious infection had an anti-S antibody level of >40,000 AU/mL, the highest observed during the first testing. She had encountered a cluster of individuals with SARS-CoV-2 infection in her workplace 3 weeks after the second vaccination and had close contact with a COVID-19 patient. It is likely that she was exposed to SARS-CoV-2, but the very high level of anti-S antibodies 3 weeks after the vaccination prevented both a positive PCR test result and symptoms, and that the exposure to the virus at that time boosted her anti-S antibody levels, which persisted for a long time. This participant was considered to have experienced an occult infection. Another participant with a positive anti-N antibody result in the first testing and without any symptoms or PCR positive results was also considered to have had an occult infection.

Several months before this study, most studies of anti-S antibody levels after vaccination had examined antibodies within 30 days after vaccination [6,7,8]. Gervain et al. [9] measured antibodies at 7, 67, and 97 days after the second BNT162b2 vaccination was administered to 47 healthcare employees and found that the levels at 67 and 97 days after vaccination were 60% and 75% lower, respectively, than the levels at 7 days after vaccination.

Recently, some studies have evaluated the anti-S antibody levels nearly 6 months after vaccination [10,11,12]. Levin et al. [10] performed a large-scale serological study in Israel that included more than 4000 healthcare employees; they performed testing every 4 weeks for 24 weeks and showed constant waning after the first sampling at 4 to 17 days after the second vaccination, with significantly lower levels in men, individuals 65 years of age or older, and those with immunosuppression. Ponticelli et al. [11] examined 162 healthcare employees within 30 days after they were administered the BNT162b2 vaccination and re-examined them six times at 30 day intervals; their results showed a constant decline in the anti-S antibody levels. Bayart et al. [12] examined 231 healthcare professionals who received the BNT162b2 vaccine for 180 days after the first vaccination and showed a constant decrease in antibody levels from 7 days after the second vaccination. They also showed a longer half-life of the antibodies in those with infection compared to that of those without infection. Our observations of 444 Japanese hospital employees are consistent with the observations of these studies.

The sex-related differences in antibody levels and body temperature after vaccination might be associated with the lower incidence of severe COVID-19 in females than in males. At our hospital, where most COVID-19 patients have severe disease, the proportion of males among all patients was 69.2%, and the proportion of males among all intubated patients was as much as 73.5%.

Sex-related differences in COVID-19 mortality have been reported in many countries. The mortality rate among patients with COVID-19 has been estimated to be 1.7 times higher for males than for females [13]. This has been attributed to biological factors such as genes in the X and Y chromosomes or sex hormones [13]. Takahashi et al. [14] conducted a study of patients with moderately severe COVID-19 and found that IL-8 and IL-18 levels were higher in males than in females, and that T-cell activation was stronger in females than in males. It is speculated that the decreased levels of testosterone in older males is associated with immune dysfunction, as well as the other complications of COVID-19 [15,16], whereas females have a stronger immune response based on genetic factors, such as Toll-like receptor 7 encoded in X chromosome, which might not be affected by aging or hormonal changes [17].

For vaccinated individuals, even after the anti-S antibody levels have decreased, the reported risk of developing severe disease is very low. A large data analysis performed in New York revealed that the overall effectiveness of a combination of three types of vaccines (BNT162b2, mRNA-1273, and Ad26.COV2.S) for preventing new laboratory-confirmed SARS-CoV-2 infection decreased from 91.7% to 79.8% between March and July 2021, but the effectiveness for preventing hospital admission was maintained at 91.9% to 95.3% [18]. According to a study of breakthrough infection conducted in Israel, most breakthrough infections in vaccinated individuals were mild or asymptomatic [19]. A mega-data analysis of 1,240,000 individuals in the United Kingdom also found a very low rate of severe disease among vaccinated infected individuals [20].

The low incidence among severe disease for vaccinated individuals could be attributable to rapid and strong humoral and cellular immune responses when vaccinated individuals are infected with SARS-CoV-2. However, it remains unknown as to how long this immunological memory endures; therefore, a third dose of the vaccine might be useful for maintaining immunity.

In Japan the fifth wave of SARS-CoV-2 infection occurred from July to September 2021, due to the delta variant. Therefore, this variant may have infected five participants in this study in August 2021, although the sequences were not confirmed. The data of 6 months efficacy in the clinical trial of the BNT162b2 vaccine [1] were obtained before the appearance of the delta variant. Thereafter, Lopez Bernal et al. [2] examined big data in United Kingdom and found slightly less efficacy of the BNT162b2 vaccine against the delta variant than against the alpha variant (88.0% and 93.7%, respectively). A real-world mega-data study in Israel [5] should have observed breakthrough infection by the delta variant because the observation period was July 2021 when the delta variant was dominant all over the world. We speculate that the threshold antibody level required for protection is approximately 1000 AU/mL, according to the results of this study and the data from Israel. The omicron variant, first discovered in southern Africa in November 2021, has been rapidly spreading worldwide since December 2021. The threshold of the antibody levels required to prevent infection should be estimated according to each variant.

This study had some limitations. First, a limited number of participants were tested within 2 months after receiving the second dose of the vaccine. Therefore, it does not provide information about anti-S antibody level patterns within 90 days after vaccination. Second, the neutralizing antibodies of participants were not tested, although the anti-S antibody and neutralizing antibody levels have been shown to strongly correlate with each other [21], we did not confirm this independently. Third, the estimation of the threshold of anti-S antibody levels of 1000 AU/mL was based on projecting our results onto data from previous studies, and therefore further study is needed to verify this estimation. Fourth, we did not collect data on physical diseases accompanying the immunocompromised state of participants. Therefore, we could not explain the low levels of antibodies in some participants.

## 5. Conclusions

Considerable decreases in anti-S antibody levels were confirmed from 100 to 200 days after the second BNT162b vaccine administration. Compared to the antibody levels 100 days after the second vaccination, more than a half of participants are thought to have lost the effectiveness to prevent infection 200 days after the second vaccination. The short lifespan of the antibodies should explain the recurrences of epidemics followed by several months of remission after nationwide vaccination. It is highly probable that a third vaccination is necessary to prevent a COVID-19 resurgence. According to the observation of a more gradual decrease in antibody levels in infected participants than in uninfected participants, anti-S antibodies induced by a third vaccination are expected to persist longer than those induced by the second vaccination. Measuring anti-S antibody levels might be a useful alternative to measuring neutralizing antibodies to assess the effectiveness of the vaccine and the need for an extra dose of the vaccine, especially among vulnerable individuals.

In December 2021, the employees in our hospital received a third dose of vaccine ahead of general population, approximately 260 days after the second vaccination.

## Data Availability

Data are contained within the article.
